# 

**Published:** 1859-12

**Authors:** 


### ORIGINAL COMMUNICATIONS.

PAGE.

ARTICLE I.—Case of Rupture of the Uterus with Operation. By R. A. T. Ridley, A. M. M. D., of LaGrange, Ga., ............ 193

ARTICLE II.—Why are we not a Healthy People? By Chas. H. Bass, M. D., Assistant Physician to the Georgia State Lunatic Asylum, Milledgeville, Ga., ............ 197

ARTICLE III.—Hygiene in the Practice of Medicine. By V. H. Taliaferro, M. D., Atlanta, Ga., ............ 270

ARTICLE IV.—A Case of Morbid Growth of the Gums? By J. P. H. Brown, Dentist, Atlanta, Ga., ............ 212

### SELECTIONS.

The Life and Labors of Laennec: An Introductory Address delivered at the New Orleans School of Medicine, November 14, 1859,... 214
Case of Abortion ............ 229
The Mouth to Mouth *vs.* The Marshall Hall Method in Asphyxia Neonatorum, ............ 232
Abdominal Tumors Mis-taken for Ovarian, ............ 237

### EDITORIAL AND MISCELLANEOUS.

A Visit to Philadelphia and New-York, ............ 241
Transactions of the American Medical Association. Vol. XII, 1859, 246
To Subscribers, ............ 247
Our Journal, ............ 247
Books, &c. Received, ............ 247
Exodus of Southern Medical Students, ............ 248
Comparative Advantages of Ether and Chloroform, ............ 250
The Age of Man, ............ 251
Medicinal Garden, ............ 252
Southern Students in Northern Medical Colleges, ............ 253
New Medical Journal, ............ 254
The New Medical School at Chicago, ............ 254
Means of Preventing the Pitting of Small Pox, ............ 255
Subterranean Fire in Belgium, ............ 256
Meteorological Table ............ 256

